# Editorial: The Developmental Seizure-Induced Hippocampal Mossy Fiber Sprouting: Target for Epilepsy Therapies?

**DOI:** 10.3389/fneur.2019.01212

**Published:** 2019-11-13

**Authors:** Hong Ni, Timo Kirschstein, Braxton A. Norwood, Ching Liang Hsieh

**Affiliations:** ^1^Division of Brain Science, Institute of Pediatric Research, Children's Hospital of Soochow University, Suzhou, China; ^2^Oscar Langendorff Institute of Physiology, University of Rostock, Rostock, Germany; ^3^Expesicor Inc., Kalispell, MT, United States; ^4^Research Center for Chinese Medicine and Acupuncture, China Medical University, Taichung, China

**Keywords:** mossy fiber sprouting, epilepsy, hippocampus, dentate gyrus, therapies

In most patients, epilepsy arises due to various initial precipitating injuries during the developmental stages. A major challenge in the neuroscience and neuroclinical fields is understanding how this precipitating injury produces a persistent reorganization of the brain's neural network, thereby transforming the normal brain into epileptogenesis. Identifying the pathological basis of epileptic seizure may lay the foundation for the development of new anti-epileptic drugs, benefiting 60 million people with epilepsy worldwide. This is why our editors chose the theme “The Developmental Seizure-Induced Hippocampal Mossy Fiber Sprouting: Target for Epilepsy Therapies?”

By using transgenic mice expressing the cell-killer gene thymidine kinase in granulosa cell progenitors or through a diphtheria toxin receptor expression strategy, newborn granule cell ablation can significantly reduce the frequency of seizures but has no effect on Mossy Fiber Sprouting (MFS) (1, 2). MFS develops independently of the loss of mossy fiber targets, and its presence is not necessarily associated with the development of spontaneous seizures [(3, 4); Cavarsan et al.]. The development of mossy fiber sprouting may be associated with epilepsy comorbidities rather than with seizure incidence. For example, people with mesial temporal lobe epilepsy (mTLE) and depression show more sprouting than those with only mTLE [(5); Godale and Danzer].

MFS is an active phenomenon, and possibly a normal adaptive mechanism that is reversible, which might be related to the replacement or restoration of lost synaptic contacts rather than to the formation of recurrent excitatory circuits in dentate granule cells [(6–8); Cavarsan et al.; Koyama and Ikegaya]. A 4-week zinc-deficient diet exacerbated MFS caused by developmental seizures, accompanied by cognitive deficits and reduced seizure thresholds. In contrast, zinc supplementation for 4 weeks significantly reduced MFS and improved the above-mentioned damage-related changes. Mitophagy-mediated zinc homeostasis via mitochondrial activation may be a potential mechanism [(9); Jin et al.; Li et al.].

This Research Topic collects seven articles: four animal studies (including one *in vitro* study) and three reviews. With respect to the demographics of this Research Topic collection, the corresponding authors are from Japan, the United States, Canada, Brazil, South Korea, and China. We hope that the information gathered from this topic will help promote post-epilepsy MFS study and help promote clinical translational medical research to better prevent and treat these injuries in the near future.

## Author Contributions

HN wrote the draft. TK, BN, and CH reviewed the manuscript.

### Conflict of Interest

BN was employed by the company Expesicor Inc. The remaining authors declare that the research was conducted in the absence of any commercial or financial relationships that could be construed as a potential conflict of interest.
